# The Safety and Health Improvement: Enhancing Law Enforcement Departments Study: Feasibility and Findings

**DOI:** 10.3389/fpubh.2014.00038

**Published:** 2014-05-08

**Authors:** Kerry S. Kuehl, Diane L. Elliot, Linn Goldberg, David P. MacKinnon, Bryan J. Vila, Jennifer Smith, Milica Miočević, Holly P. O’Rourke, Matthew J. Valente, Carol DeFrancesco, Adriana Sleigh, Wendy McGinnis

**Affiliations:** ^1^Department of Medicine, Oregon Health & Science University, Portland, OR, USA; ^2^Department of Psychology, Arizona State University, Tempe, AZ, USA; ^3^Department of Criminal Justice & Criminology, Washington State University Spokane, Spokane, WA, USA

**Keywords:** health promotion, safety, law enforcement, occupational health, team-based

## Abstract

This randomized prospective trial aimed to assess the feasibility and efficacy of a team-based worksite health and safety intervention for law enforcement personnel. Four-hundred and eight subjects were enrolled and half were randomized to meet for weekly, peer-led sessions delivered from a scripted team-based health and safety curriculum. Curriculum addressed: exercise, nutrition, stress, sleep, body weight, injury, and other unhealthy lifestyle behaviors such as smoking and heavy alcohol use. Health and safety questionnaires administered before and after the intervention found significant improvements for increased fruit and vegetable consumption, overall healthy eating, increased sleep quantity and sleep quality, and reduced personal stress.

## Introduction

In the U.S., close to 800,000 individuals work in law enforcement (1). Charged with the critical work of protecting the population’s safety, these individuals have unique health issues that impact their well being, longevity, and, importantly, their job performance and safety (2, 3). Certain law enforcement work-related issues are well recognized, such as the importance of physical abilities in apprehending suspects; shift work including long work hours; and the ever present psychological stress of law enforcement work (4–8).

Additionally, law enforcement personnel are a high-risk group for musculo-skeletal injuries, fatigue/stress-related disorders, sleep disorders, metabolic syndrome, and cardiovascular disease (9–11). Overall, the life expectancy of those working in law enforcement is between 6 and 15 years less than the average American (12–16). Although recent research has highlighted elevated risk factors and corresponding health outcomes among this population, evidence-based occupational safety and health programs for law enforcement workers are lacking.

Law enforcement work is characterized by tight knit groups of colleagues who rely on each other for safety. This work structure and organization culture is suited for a team-based health promotion program comprised of colleagues working the same shift or fulfilling similar work roles. We have demonstrated success impacting health attitudes and behaviors through other team-based health promotion programs for athletic teams and firefighters (17–19).

The safety and health improvement: enhancing law enforcement departments (SHIELD) study is sponsored by the Centers for Disease Control and Prevention and the National Institute for Occupational Safety and Health to investigate strategies to promote health and safety of law enforcement personnel. SHIELD addresses modifiable factors that synergize to adversely impact law enforcement personnel’s health, safety, and work performance: high stress, sleep deprivation, lack of physical abilities, and unhealthy lifestyles (20–24). The modifiable lifestyle factors addressed in the SHIELD program included: healthy diet, daily physical activity, achieve and maintain optimal body weight, reduce stress, increase sleep, decrease tobacco and heavy alcohol use.

The objective of the SHIELD study was to test the feasibility and potential efficacy of this team-based intervention approach. We report the trials’ 6-month findings. We hypothesized that law enforcement workers’ health and occupational safety would be improved with a peer-led, team-based health promotion and protection program.

## Materials and Methods

### Study population and recruitment

One police department and two sheriff’s offices from Oregon and southwest Washington were identified and recruited for participation in this study. The two sheriff’s offices were recruited due to similar size, staffing patterns, and scheduling. The police department was recruited due to its natural division of staff into two precincts.

Partnering law enforcement organizations were one police department, which at the time of study recruitment employed approximately 200 sworn and 35 civilian staff; a sheriff’s office employing approximately 246 sworn officers and 106 civilian staff; and a sheriff’s office with 244 sworn officers and 97 civilian staff. A total of 408 individuals from across the participating organizations consented to participate in the 2-year study. The Institutional Review Board of the Oregon Health & Science University first approved the study in April of 2010.

### Randomization

Within each of these agencies, study participants were organized into teams. Teams consisted of three to seven persons who work together at the same or similar jobs. Most teams were naturally occurring (e.g., patrol officers on the same shift), and some teams were assigned based on similar job description and work location (e.g., various support staff at a single location). Teams were then organized into blocks based on shift, schedule, and physical work location. Each block consisted of one to six teams. The blocks were organized into matched pairs: patrol blocks were matched with patrol blocks, jail staff blocks matched with jail staff blocks, training unit blocks matched with training unit blocks, and remaining blocks matched in a similar manner. Blocks were used for matching in the randomization only, and had no bearing on program delivery. The formation of blocks reduced the risk of contamination between teams.

This matching scheme organized the 86 teams into 42 blocks, or 21 matched pairs of blocks. After the blocks were randomized, 204 participants from 45 teams in 21 blocks were in the treatment condition and 204 participants from 41 teams in 21 blocks were in the control condition.

**Figure 1:**
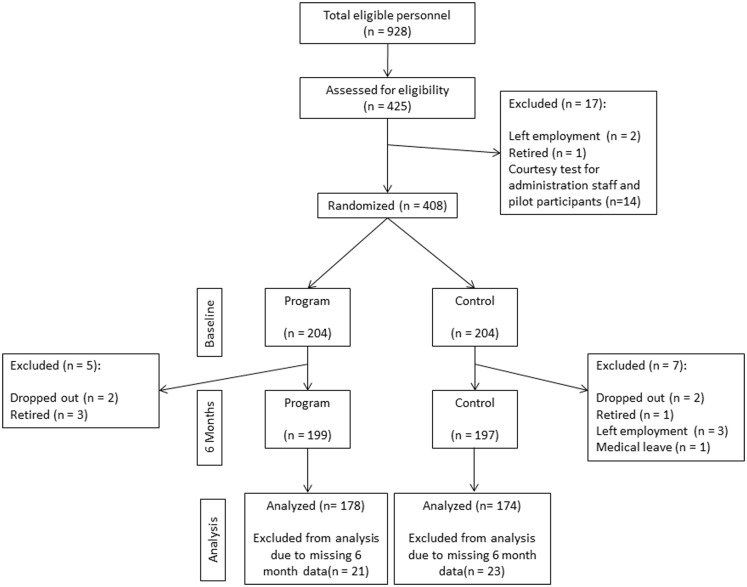
**Participant enrollment and outcomes**.

For the three departments, of the 928 eligible employees, 408 recruited individuals met inclusion criteria, and the allocation, follow-up, and assessment numbers are presented in Figure 1.

### Measures

All study participants received comprehensive medical screening at baseline (results not presented here). As part of the intervention assessment, study participants completed a written survey prior to randomization and approximately 6 months after initial testing. The survey instrument included information about demographics, position, years of service, and work schedule. Items were assessed using constructs with established reliability from our previous studies (25), supplemented by items/constructs from published instruments. Scale components with correlations between baseline and follow-up are shown in Table 1. More information on the scales is available as part of a separate addendum, to be located online via http://ripl.faculty.asu.edu/

**Table 1:** **Scales and test–retest correlations**.

Survey items and constructs	Test–retest correlation	
**NUTRITION**		
Fruit and vegetable consumption was assessed using the National Cancer Institute’s fruit and vegetable all day screener, a standardized instrument of daily servings (26). Additional questions were added to assess frequency and amount of dietary fat	Fruit	0.485
	Veg	0.480
	Fruit and veg	0.554
	Fat	0.696
**SLEEP/FATIGUE**		
Subjective, self-reported measures of sleep and fatigue were assessed using items from the National Institutes of Health Patient-Reported Outcomes Information System (PROMIS) sleep disturbance, the Pittsburgh Sleep Quality Index (PSQI), and the Karolinska Sleepiness Scale (27–29)	Sleep quality	0.652
	Sleep quantity	0.652
	Fatigue	0.645
	Karolinska	0.292
**HEALTH PERCEPTIONS**		
General health status was assessed with the SF-36 (30)	SF-36	0.743
**MUSCULO-SKELETAL DISCOMFORT**		
Musculo-skeletal discomfort was assessed using 18 of the 54-items of the Cornell Musculo-skeletal Discomfort Questionnaire (CMDQ) (31)	Musculo-skeletal pain	0.635
**STRESS, HEALTHY EATING, AND PHYSICAL ACTIVITY**		
Constructs with established reliability from our previous studies were used (25)	Stress	0.732
	Healthy eating	0.733
	Physical activity	0.632
**BURNOUT**		
Burnout was assessed with questions from the emotional exhaustion subscale of the Maslach Burnout Inventory (MBI) (32)	Burnout	0.654

### Intervention

Each team received a team box containing the materials needed for the program. Teams consisted of individuals who worked in the same location or type of work on the same shift schedule. One participant from each intervention team was self-selected to serve as team leader. Team leaders received a 20-min orientation to the program and a scripted team leader manual to guide each session. Other team members used corresponding workbooks. The curriculum included 12 30-min, scripted, peer-led, team-based sessions. Sessions were scheduled weekly during work hours. Each session comprised of three or four brief interactive activities about healthy eating, exercise, body weight, stress, sleep, and other lifestyle factors. Language throughout the scripted sessions emphasized the team social support aspect of the program. The peer-led, team format encouraged accountability as well as fostering support at the worksite. Participants were encouraged to check in with one another about the weekly goals and scripted discussion prompts elicited the sharing of suggestions and tips.

Research staff observed a random selection of approximately one-third of the sessions, taking notes on participant attendance, engagement, and fidelity using the scripted lesson plans as behavioral checklists.

### Control

Individuals assigned to teams that were randomized to the control condition participated in the same baseline health assessment and completed the same questionnaires as intervention individuals. Results of the baseline medical screening were communicated to all subjects with appropriate medical advice and follow-up for abnormal test results with their physician. No other health promotion programs or educational materials were provided to control participants.

### Statistical analysis

The statistical analysis incorporated the nesting of individual subjects within the 86 teams. The intraclass correlations for teams at baseline ranged from 0.000 for the Karolinska Sleepiness Scale to 0.052 for total fruit and vegetable consumption. Missing data at the 6-month follow-up were treated as missing at random by including baseline measures in the statistical analysis. This multilevel regression model was estimated using the SAS mixed program using a model for two repeated measures from each subject, clustering of groups of subjects in teams and observations missing at random. More information on the analysis is available as a separate addendum, to be located online via http://ripl.faculty.asu.edu/

Intervention significance tests were assessed in the multilevel analyses in a repeated measures model where the interaction of condition and time provided the test of the intervention. This group-by-time interaction indicates whether the treatment and control groups changed differently over time for each construct, and thus represents the program effect. Statistical significance with *p*-values are presented in Table 3 and discussed in Section “Program Effects” below.

## Results

Results from this final model are based on repeated measures from participants, participants in teams, and the use of all data including data from participants only measured at baseline. Additional analyses based solely on individuals or solely on team means generally led to the same research conclusions.

### Adherence to format and acceptability

Fifty-six of the 408 participants did not complete follow-up at 6-month testing. Of the 56 participants who did not complete post testing, 26 were randomized to the treatment group and 30 were randomized to the control group. For the 37.6% of the sessions observed, the average session length was 31.4 ± 11 min (mean ± SD), and 97% of the scripted content was delivered. During sessions that were observed, the average attendance at the team sessions was 87%. The curriculum was delivered with high fidelity and no other health promotion programs were initiated during the study period.

### Demographics

Selected demographics for participants are shown in Table 2, separated by condition. The groups were approximately the same at baseline.

**Table 2:** **Descriptive demographic variables at baseline (number or mean [SD])**.

Variable	Control	Treatment
Age (years)^a^	41.6 (9.37)	44.3 (9.67)
Gender
Male	140	114
Female	64	90
Ethnicity
White	184	187
Black/African American	1	5
Asian	3	2
Native Hawaiian/other Pacific Islander	4	1
American Indian/Native Alaskan	2	1
Other	8	7
Marital status
Married	135	155
Divorced	36	25
Widowed	1	7
Separated	1	3
Never married	20	6
Member of an unmarried couple	8	6
Time in law enforcement (years)	13.5 (7.67)	14.7 (8.89)

*^a^ Treatment significantly different from control when tested at individual level, but not significant when accounting for clustering within teams*.

Participants in the control group were younger than participants in the treatment group when tested at the individual level but not when accounted for clustering in teams. Given the random assignment of teams to conditions, the statistical test for baseline equivalence in the multilevel model is a more accurate test of baseline equivalence. As shown in Table 3, constructs did not differ at baseline with the exception of healthy eating self construct only. Participants in the treatment group rated themselves as healthier eaters than participants in the control group (*p* = 0.0355).

**Table 3:** **Program effects assessed at 6 months [observed means (SD) and predicted means]**.

Variables	Control group				Intervention group					
	Baseline		6-month follow-up		Baseline		6-month follow-up			*p*
	Observed mean score	Predicted mean score	Observed mean score	Predicted mean score	Observed mean score	Predicted mean score	Observed mean score	Predicted mean score	Program effect size	
Fruit consumption^a^	2.18 (1.12)	2.19	2.03 (1.47)	2.12	2.07 (1.02)	2.06	3.05 (1.50)	3.09	0.50	<0.0001^a^
Vegetable consumption^a^	3.74 (2.26)	3.68	3.64 (1.81)	3.56	3.53 (1.29)	3.57	4.40 (1.52)	4.46	0.30	0.004^a^
Fruit/vegetable consumption^a^	5.92 (3.07)	5.87	5.67 (2.64)	5.68	5.60 (1.94)	5.61	7.48 (2.33)	7.55	0.47	<0.0001^a^
Fat consumption	−0.08 (0.39)	−0.04	0.14 (0.38)	0.07	0.07 (0.43)	0.04	0.35 (0.47)	0.18	0.08	0.267
Sleep quality^a^	0.03 (0.83)	0.004	−0.01 (0.80)	0.02	−0.02 (0.96)	−0.01	0.57 (0.88)	0.29	0.32	0.0001^a^
Sleep quantity^a^	−0.09 (0.94)	−0.03	−0.06 (0.85)	−0.02	0.08 (0.74)	0.03	0.52 (0.92)	0.26	0.26	0.004^a^
Karolinska Sleepiness Scale	5.87 (0.83)	5.92	5.42 (0.98)	5.41	5.99 (0.84)	5.95	5.68 (1.23)	5.74	0.17	0.181
Fatigue	−0.14 (0.70)	−0.06	−0.13 (0.71)	−0.05	0.12 (0.70)	0.07	0.26 (0.70)	0.16	0.11	0.192
SF-36: general health^b^	3.36 (0.26)	3.59	3.39 (0.24)	3.42	3.30 (0.26)	3.55	3.47 (0.23)	3.48	0.14	0.083
Musculo-skeletal pain	1.87 (0.07)	1.86	1.85 (0.09)	1.85	1.88 (0.07)	1.88	1.86 (0.09)	1.87	0.00	0.795
Musculo-skeletal pain with foot pain	1.87 (0.07)	1.86	1.85 (0.09)	1.85	1.88 (0.06)	1.88	1.87 (0.09)	1.87	0.00	0.932
Stress self^a^	4.07 (0.63)	4.05	4.11 (0.75)	4.04	4.14 (0.64)	4.12	4.37 (0.66)	4.32	0.16	0.034^a^
Healthy eating self^a^	−0.19 (0.74)	−0.08	0.22 (0.74)	0.12	0.17 (0.79)	0.08	0.82 (0.79)	0.44	0.21	0.009^a^
Physical activity self	−0.10 (0.76)	−0.03	0.39 (0.99)	0.21	0.08 (0.87)	0.03	0.51 (0.90)	0.30	0.04	0.641
Burnout	3.94 (0.67)	3.94	3.99 (0.73)	3.92	4.11 (0.57)	4.12	4.05 (0.79)	4.07	−0.02	0.772
Tobacco use^b^	3.53 (0.41)	3.52	3.56 (0.41)	3.52	3.32 (0.57)	3.29	3.38 (0.60)	3.37	0.08	0.070
Alcohol consumption^b^	−0.02 (0.69)	−0.02	0.00 (0.79)	−0.02	0.02 (0.63)	0.03	0.09 (0.90)	0.08	0.07	0.105
Depression^b^	4.92 (0.53)	5.22	4.76 (0.69)	5.09	4.99 (0.61)	5.36	5.03 (0.80)	5.40	0.14	0.093

*^a^ Indicates statistically significant program effect of group by time *p* < 0.05*.

*^b^ Indicates marginally significant program effect of group by time *p* < 0.10*.

### Program effects

Program effects for each construct were estimated using a multilevel model, including all individuals nested within one of the 86 participating teams. Table 3 shows the program effects with means for treatment and control groups at baseline and after 6-month follow-up. Program effect size estimates are given in the far right column on Table 3. The effect size measure was obtained by taking the difference in the change in program and control groups, and then standardized by dividing by the pooled standard deviation across individuals at baseline.

Significant program effects were observed for many program outcomes. Program effects were observed for fruit consumption (*p* < 0.0001), vegetable consumption (*p* = 0.004), combined fruit and vegetable consumption (*p* < 0.0001), sleep quality (*p* = 0.0001) and quantity (*p* = 0.004), stress (*p* = 0.03), and healthy eating (*p* = 0.009). A marginally significant program effect was found for the SF-36 measures of general health (*p* = 0.08), alcohol use (*p* = 0.10), tobacco use (*p* = 0.07), and depression (*p* = 0.09). Notably, other major construct outcome variable effects were in the correct direction, though non-significant, including physical activity, fatigue, and dietary fat consumption. No beneficial effects on burnout or musculo-skeletal pain were obtained.

## Discussion

A combined approach integrating health promotion with health protection delivered through a scripted, team-based curriculum positively impacted health and safety outcomes of law enforcement personnel in the areas of nutrition, sleep, stress, and with positive trends observed to reduce tobacco use and heavy alcohol consumption, and reduce depression. The high session attendance rate of 87% suggests that the team-based intervention program was well-received and feasible in our law enforcement departments.

It is well documented that law enforcement officers (LEO’s) experience an increased prevalence of cardiovascular disease due in part to elevated diabetes, hypertension, and hypercholesterolemia. However, other characteristics of law enforcement work likely play a role in these elevated health risks including increased stress and sleep disorders. Recent published results found 40% of surveyed police officers had symptoms consistent with a sleep disorder (8). Sleep disorders and deficiencies are both a health and safety risk for those in law enforcement (5) and may be a contributing factor to adverse health and safety outcomes. It is interesting to note that those in the intervention arm of this study, who went through the 12-week worksite health and safety curriculum, reported a statistically and clinically significant increase in both sleep quality and sleep duration and it occurred for personnel who worked on day, swing, and night shift hours. This is important as one study suggested that graveyard (or night shift) workers had increased prevalence of metabolic syndrome and sleep disorder than those LEO’s on day shifts (8, 9). By improving sleep quality and quantity among this population, we would expect a similar reduction in work-related illness and injury due to the known association of sleep deprivation and injuries.

Similar to the high prevalence of sleep disorders among LEO’s, there is a high prevalence of stress and stress-related issues among LEO’s. Due to the nature of the job, the law enforcement profession is recognized as particularly stressful with high rates of stress-related mental health issues and suicide (1, 7, 11, 20). This stress is associated with other adverse health outcomes, particularly the high risk of cardiovascular disease, faced by those who work in law enforcement (12, 13, 24). Our SHIELD occupational health and safety program had three specific stress reduction modules designed to engage participants to use these stress reducing activities on a daily basis. Self-reported stress among the intervention group was significantly lower as compared to the control group at 6 months. This suggests that our team-based, peer-led curriculum was effective at reducing personnel stress by targeting specific stress relieving healthy actions that LEO’s could implement easily both on a routine daily basis and during a crisis.

Similar to our firefighter wellness program (19), significant dietary changes were observed in several areas. Intervention participants increased fruit consumption, and vegetable consumption by two servings per day. We know that increasing fruit and vegetable intake by 1/2 serving per day is clinically important to reduce cardiovascular disease and certain cancers (19). A statistically significant increase of two servings per day has the potential to markedly reduce chronic disease over time. It is well documented that law enforcement personnel have increased cardiovascular disease, metabolic syndrome, and certain cancer types as compared to U.S. adults, and these may in part be related to poor dietary habits of law enforcement personnel. These types of fruit and vegetable changes were observed in our previous PHLAME study among firefighters (19, 33, 34). The SHIELD program targeted specific dietary behavior change activities with daily and weekly goals that encouraged helping each other achieve healthy nutrition alternatives during the shift and at home. These activities included a fast food makeover session on how to replace an unhealthy meal with a healthy meal, how to shop and cook low-fat, how to reduce calories in snacks, what to bring in your lunch from home, a session called “brownbag makeover.” These were all team-based activities and only took 30 min to complete while at work for a total of 12 weeks that capitalize on the power of the team to create a healthy culture both at work and home.

### Limitations

Since the intervention was not limited to one site, there was a risk of contamination from intervention subjects communicating program content to control subjects. Randomization by blocks based on physical work location and shift attempted to reduce this spill over. Any communication of program content across teams who did and did not receive the intervention program would have reduced the observed program effect. Further, the randomization within sites precluded adding a work environment component, such as posters or stair reminders, which are frequently a component of worksite safety and wellness programs. This limitation also makes the program effects more striking. Our findings should be generalized with caution outside of this study. Participation in the study was voluntary and may have resulted in the recruitment of individuals who were more motivated to change than the average law enforcement personnel. However the experimental design and relatively high participation enhance the validity of our findings.

## Conclusion

Law enforcement personnel are a high-risk group for occupational injuries and illnesses, resulting in a life expectancy less than the average American (7–11). Few law enforcement organizations have health promotion/harm reduction programs, despite a demonstrated need and predictions that occupational wellness is a critical component of recruiting and maintaining an effective workforce. This study demonstrates that a team-based, peer-led scripted health promotion program, and protection for law enforcement personnel incorporated into their daily work routine is both feasible and may be ideally suited to harness positive peer pressure to improve the health and safety of law enforcement personnel. While many studies among law enforcement workers highlight the adverse health outcomes faced by those in this profession (9–16, 20), this study is promising in that it suggests that tailored lifestyle and behavior change interventions could counter the adverse health effects associated with work in law enforcement.

## Conflict of Interest Statement

The PHLAME program is listed on the Cancer Control P.L.A.N.E.T. website of evidence-based programs, and the PHLAME TEAM program is distributed through the Center for Health Promotion Research at Oregon Health & Science University (OHSU). OHSU and Drs. Kuehl, Elliot, and Goldberg have a financial interest from the commercial sale of technologies used in this research. This potential conflict of interest has been reviewed and managed by the OHSU Conflict of Interest in Research Committee. The remaining authors declare no conflicts of interest.

## Supplementary Material

The Supplementary Material for this article can be found online at http://www.frontiersin.org/journal/10.3389/fpubh.2014.00038/abstract
