# Supplementary material for: The Safety and Health Improvement: Enhancing Law Enforcement Departments Study: Feasibility and Findings
Source: Front Public Health. 2014 May 8;2:38. doi: 10.3389/fpubh.2014.00038 (PMC4021110; doi:10.3389/fpubh.2014.00038)
Supplement: Supplementary file 1 [file Data_Sheet1.DOCX]

From: Dr Kerry S Kuehl, Principal Investigator and Corresponding Author

To: Readers of the SHIELD Feasibility and Findings paper

Re: Multilevel SHIELD Analyses and Effect Size Calculation

Date: 4/7/14

A multilevel model was used to evaluate the SHIELD program to account for the individual-level and team-level effects that exist within the data. Because team members are likely to share characteristics, they are more likely to respond in the same way compared to individuals not on their team. The equation for the two-level multilevel model for the effect of X on Y is as follows:

$$Y_{ij}=\gamma_{0}+b_{1}X_{j}+u_{0j}+e_{ij}$$

where $Y_{ij}$ is the individual-level outcome score, $\gamma_{0}$ is the grand mean, $b_{1}$ is the slope relating the independent variable X at level 2 to the group-level intercept(s), $u_{0j}$ is the error component for the deviation of the group intercepts from the overall intercept, and $e_{ij}$ is the individual-level random error.

The evaluate of the six-month SHIELD program effects requires a three-level multilevel model to account for the repeated measures (two time points) within individuals, and individuals within clusters (or “teams”), as well as an interaction between group (treatment/control) and time (time 1/time2) to produce the program effects for a given outcome. An example equation for the three-level multilevel program effect on fruits and vegetables is as follows:

$$FV_{ijk}=\gamma_{0}+\gamma_{1}(time_{ijk})+\gamma_{2}(group_{j})+\gamma_{3}(time_{ijk})(group_{j})+u_{0k}+u_{1k}(time_{ijk})+u_{0jk}+e_{ijk}$$

where $FV_{ijk}$ is the level-1 fruits and vegetables outcome, $\gamma_{0}$ is the grand mean, $\gamma_{1}$ is the slope relating level-1 time to level-1 fruits and vegetables, $\gamma_{2}$ is the slope relating level-3 group to level-1 fruits and vegetables, $\gamma_{3}$ is the slope representing the interaction between level-1 time and level-3 group on level-1 fruits and vegetables, $u_{0k}$ and $u_{1k}$ represent team-level differences (allowing intercepts and slopes to differ at team-level), $u_{0jk}$ represents individual differences (allowing intercept to differ at individual-level, but not slope), and $e_{ijk}$ represents individual-level random error. A univariate data set was used so that all observations were used in the analysis including data from participants who were measured at only baseline. The analysis assumes that the data are Missing at Random.

Below is example syntax of the proc mixed statements used to assess program effects on continuous variables that adjusts for individuals clustered in teams. The outcome in the example given is fruits and vegetables. A description of each line is given below the syntax.

*1; TITLE "FRUIT/VEGETABLE CONSUMPTION (ADJUSTED FOR CLUSTERING IN TEAMS)";

*2; OPTIONS PS=**59** LS=**80** CENTER NODATE NONUMBER;

*3; **PROC** **MIXED** DATA=SHIELD2 NOCLPRINT covtest method=ml;

*4; CLASS GROUP ID TIME TEAM;

*5; MODEL FRTVEG=GROUP TIME GROUP*TIME/solution ddfm=sat;

*6; RANDOM INTERCEPT/SUBJECT=TEAM TYPE=UN;

*7; RANDOM INTERCEPT/SUBJECT=ID(TEAM) TYPE=UN;

*8; LSMEANS GROUP*TIME/SLICE=GROUP SLICE=TIME CL E DIFF=ALL;

*9; **RUN**;**QUIT**;

Line 1: Gives the analysis title.

Line 2: Sets output formatting.

Line 3: This invokes the MIXED procedure in SAS, specifies the data file to be used, asks SAS not to print class-level information (noclprint), has SAS show hypothesis tests for the variance and covariance parts of the model (covtest), and specifies that the model should be estimated using maximum likelihood (method=ML).

Line 4: The class statement contains the levels at which individuals are clustered as well as the categorical independent variables (group and time).

Line 5: The model statement allows us to specify the single dependent variable to be used, fruits and vegetables, and the fixed effects to be estimated (model frtveg=group time group*time), produces a solution for the fixed effects (solution), and uses the Satterthwaite method for computing degrees of freedom (ddfm=sat).

Line 6: The random statement defines the random effects in the model. This statement (random intercept) specifies the intercept as a random effect at team-level (subject=team) and specifies an unstructured covariance matrix (type=un).

Line 7: This random statement specifies a random intercept for time (random time) at individual-level (subject=id(team)) and specifies an unstructured covariance matrix (type=un).

Line 8: The lsmeans statement computes least squares means for the fixed effects, also known as the marginal means, for the interaction effect of group by time (lsmeans group*time). We then partition the effects to look at the means of group at each level of time, and the means of time at each level of group (slice=group slice=time), construct confidence limits for means (CL), print the coefficient matrix associated with the least squares mean (E), and display the differences of the least squares means (diff=all).

Line 9: Runs the procedure.

Below is a set of syntax that produces covariance parameter estimates, fixed effect estimates, and *p* values that are comparable to the syntax above, but parameterizes the model in a slightly different way.

TITLE " FRUIT/VEGETABLE CONSUMPTION (ADJUSTED FOR CLUSTERING IN TEAMS)"

OPTIONS PS=**59** LS=**80** CENTER NODATE NONUMBER;

**PROC** **MIXED** DATA=SHIELD2 NOCLPRINT info covtest;

CLASS GROUP ID TIME TEAM;

MODEL FRTVEG=GROUP TIME GROUP*TIME/solution ddfm=sat;

REPEATED time/type=cs sub=id(team*group) r rcorr;

RANDOM INTERCEPT /SUBJECT=team(group) TYPE=UN;

LSMEANS GROUP*TIME/SLICE=GROUP SLICE=TIME CL E DIFF=ALL;

**RUN**;**QUIT**;

Effect Size Calculations

The standardized effect size measures for the program effect in the paper were computed following formulas from Morris (2008). Because the effect size is standardized it can be interpreted in a similar fashion as Cohen’s d. The following formula was used:

$d=\frac{\left[ {(M}_{post,T}-M_{pre,T} \right)-(M_{post,C}-M_{pre,C})]}{{SD}_{pre}}$, $where {SD}_{pre} is the pooled standard deviation across treatment and control groups at basline.$

Morris (2008) proposes multiplying this effect size by correction factor that applies for small sample sizes. This correction factor was not used in the computation of the effect sizes for the program effects in the SHIELD 6 month paper because it was nearly equal to 1 (its value was .991) because of the relatively large sample of the SHIELD data.

The means used in the computation of this effect size measure were obtained from the lsmeans command using the Proc Mixed multilevel model. The pooled standard deviations were computed by looking at the standard deviation at baseline across the individuals as reported in the article and also for the 86 teams as described below. That is, the standard deviation is based on the individual level in the article. We felt that this was more conservative because the effect sizes with individual level data were smaller than effect sizes with team level data.

Morris, S. B. (2008). Estimating effect sizes from pretest-posttest-control group designs. *Organizational Research Methods*. 11(2), 364-386.

Special Analysis Issues

The tobacco dependent variable was an ordinal measure. We report analysis treating this variable as a continuous measure because a multilevel model described above that treated this variable as ordinal did not converge. Models based on only individual or team level data were generally consistent with the results reported in the paper.

Table 1 below contains the standardized effect size measures for the variables in the SHIELD 6 month results paper using the pooled standard deviations at the individual level. Table 2 contains the standardized effect size measures for the variables in the SHIELD 6 month results paper using the pooled standard deviations at the team level. The standardized effect size estimates computed using the pooled standard deviation at the individual level are smaller than are the standardized effect size estimates computed using the pooled standard deviation at the team level. This is because there is more variability for each variable at the individual level than there is at the team level hence higher pooled standard deviations.

Table 1

|  | Control group mean diff |  | intervention group mean diff |  | Int-Cntrl |  | SD Bpooled | Stand Diff |
| --- | --- | --- | --- | --- | --- | --- | --- | --- |
| FRUIT | -0.07 |  | 1.03 |  | 1.1 |  | 2.198348 | 0.500376 |
| VEG | -0.12 |  | 0.89 |  | 1.01 |  | 3.359023 | 0.300683 |
| FRUIT/VEG | -0.19 |  | 1.94 |  | 2.13 |  | 4.531261 | 0.470068 |
| FAT | 0.11 |  | 0.14 |  | 0.03 |  | 0.394867 | 0.075975 |
| SLEEP QUALITY | 0.016 |  | 0.3 |  | 0.284 |  | 0.889957 | 0.319117 |
| SLEEP QUANTITY | 0.01 |  | 0.23 |  | 0.22 |  | 0.853089 | 0.257886 |
| KAROLINSKA | -0.51 |  | -0.21 |  | 0.3 |  | 1.740927 | 0.172322 |
| FATIGUE | 0.01 |  | 0.09 |  | 0.08 |  | 0.714902 | 0.111903 |
| SF36: GENH | -0.17 |  | -0.07 |  | 0.1 |  | 0.720125 | 0.138865 |
| MUSPN | -0.01 |  | -0.01 |  | 0 |  | 0.170062 | 0 |
| MUSPN FOOT | -0.01 |  | -0.01 |  | 0 |  | 0.16388 | 0 |
| STRESS SELF | -0.01 |  | 0.2 |  | 0.21 |  | 1.302087 | 0.16128 |
| HEALTHY EATING | 0.2 |  | 0.36 |  | 0.16 |  | 0.764538 | 0.209277 |
| PHYSICAL ACTIVITY | 0.24 |  | 0.27 |  | 0.03 |  | 0.830902 | 0.036105 |
| BURNOUT | -0.02 |  | -0.05 |  | -0.03 |  | 1.300562 | -0.02307 |
| TOBACCO | 0 |  | 0.08 |  | 0.08 |  | 1.020912 | 0.078361 |
| ALCOHOL | 0 |  | 0.05 |  | 0.05 |  | 0.68405 | 0.073094 |
| DEPRESSION | -0.13 |  | 0.04 |  | 0.17 |  | 1.20397 | 0.141199 |

Table 2

|  | Control group mean diff |  | intervention group mean diff |  | Int-Cntrl |  | SD Bpooled | Stand Diff |
| --- | --- | --- | --- | --- | --- | --- | --- | --- |
| FRUIT | -0.07 |  | 1.03 |  | 1.1 |  | 1.072434 | 1.025704 |
| VEG | -0.12 |  | 0.89 |  | 1.01 |  | 1.815486 | 0.556325 |
| FRUIT/VEG | -0.19 |  | 1.94 |  | 2.13 |  | 2.539168 | 0.838857 |
| FAT | 0.11 |  | 0.14 |  | 0.03 |  | 0.415069 | 0.072277 |
| SLEEP QUALITY | 0.016 |  | 0.3 |  | 0.284 |  | 0.897668 | 0.316375 |
| SLEEP QUANTITY | 0.01 |  | 0.23 |  | 0.22 |  | 0.839754 | 0.261981 |
| KAROLINSKA | -0.51 |  | -0.21 |  | 0.3 |  | 0.835269 | 0.359166 |
| FATIGUE | 0.01 |  | 0.09 |  | 0.08 |  | 0.698897 | 0.114466 |
| SF36: GENH | -0.17 |  | -0.07 |  | 0.1 |  | 0.261479 | 0.38244 |
| MUSPN | -0.01 |  | -0.01 |  | 0 |  | 0.06867 | 0 |
| MUSPN FOOT | -0.01 |  | -0.01 |  | 0 |  | 0.065872 | 0 |
| STRESS SELF | -0.01 |  | 0.2 |  | 0.21 |  | 0.637245 | 0.329543 |
| HEALTHY EATING | 0.2 |  | 0.36 |  | 0.16 |  | 0.769025 | 0.208056 |
| PHYSICAL ACTIVITY | 0.24 |  | 0.27 |  | 0.03 |  | 0.823311 | 0.036438 |
| BURNOUT | -0.02 |  | -0.05 |  | -0.03 |  | 0.618184 | -0.04853 |
| TOBACCO | 0 |  | 0.08 |  | 0.08 |  | 0.504294 | 0.158637 |
| ALCOHOL | 0 |  | 0.05 |  | 0.05 |  | 0.65847 | 0.075934 |
| DEPRESSION | -0.13 |  | 0.04 |  | 0.17 |  | 0.585252 | 0.290473 |
