# Supplementary material for: The Safety and Health Improvement: Enhancing Law Enforcement Departments Study: Feasibility and Findings
Source: Front Public Health. 2014 May 8;2:38. doi: 10.3389/fpubh.2014.00038 (PMC4021110; doi:10.3389/fpubh.2014.00038)
Supplement: Supplementary file 2 [file Data_Sheet2.DOCX]

From: Dr Kerry S Kuehl, Principal Investigator and Corresponding Author

To: Readers of the SHIELD Feasibility and Findings paper

Re: SHIELD scale and index information

Date: 4/7/14

Summary

This report gives specific information on how scales and indices were constructed for the 6-month Shield project. Table 1 contains the scale/index and the items used to make the scale/index. The scale or index is written in bold. After each scale or index name, the items that were used to make the scale are listed. Table 2 lists the specific questions and response options for each scale/index. Table 3 lists the coefficient alpha for each scale. The coefficient alpha reliability measure is not appropriate for indices. The fruits and vegetables measures are treated as an index rather than a scale because individual items are added to measure amount of fruits and vegetables consumed rather than a scale where each item is treated as a measure of a latent variable.

Fruit and Vegetable Intake. The NCI Fruit and Vegetable Screener (Thompson et al., 2002) was used to measure daily nutrition behaviors in the form of fruit and vegetable consumption (available at http://appliedresearch.cancer.gov/diet/screeners/fruitveg/bymeal.pdf). The screener measures the frequency with which respondents ate 10 food items, along with the portion size of these items, over the past month. Total fruit and vegetable intake was used for this study, which represents a daily combined average of fruit and vegetable servings.

Fat Intake. The NCI Fat Screener was used to measure daily nutrition behaviors in the form of fat consumption. The screener measures the frequency with which respondents ate 14 food items, along with the portion size of these items, over the past month.

Sleep Quality. Sleep quality was assessed using four self-reported measures with items from the Pittsburgh Sleep Quality Index (available at <http://www.sleep.pitt.edu/includes/showFile.asp?fltype=doc&flID=2532>) and the National Institutes of Health Patient-Reported Outcomes Information System (PROMIS) sleep disturbance (information available at <http://www.nihpromis.org/Measures/domainframework1>). Participants were asked about quality of sleep and satisfaction with sleep as well as trouble sleeping. Cronbach’s alphas ranged from .908 to .914 across the two waves.

Sleep Quantity. Sleep quantity was assessed using three self-reported measures with items from the Pittsburgh Sleep Quality Index and the National Institutes of Health Patient-Reported Outcomes Information System (PROMIS) sleep disturbance. Participants were asked to provide the amount of hours slept on average in the past month as well as how often they felt they had gotten enough sleep in the past week. Cronbach’s alphas ranged from .811 to .820 across the two waves.

Fatigue. Fatigue was assessed using four self-reported measures with items from the Pittsburgh Sleep Quality Index and the National Institutes of Health Patient-Reported Outcomes Information System (PROMIS) sleep disturbance. Participants were asked question about feeling sleepy or exhausted, recovering energy fully between shifts, and having reserve energy when necessary. Cronbach’s alphas ranged from .687 to .711 across the two waves.

General Health. Participants’ self-reported general health was measured with the ‘General Health’ subscale of the SF-36 (Ware, Snow, Kosinski, & Gandek, 1993). This widely used subscale measures respondents’ perceptions of their own general health in relation to others. Cronbach’s alphas ranged from .764 to .770 across the two waves.

Musculoskeletal Pain (Without Feet). Musculoskeletal discomfort was assessed using 18 of the 54 items of the Cornell Musculoskeletal Discomfort Questionnaire (CMDQ) (31). Participants were asked to rate how much ache, pain, or discomfort interfered with work in the last week for 18 body parts. Cronbach’s alphas ranged from .774 to .802 across the two waves.

Musculoskeletal Pain (With Feet). Musculoskeletal discomfort was assessed using 20 of the 54 items of the Cornell Musculoskeletal Discomfort Questionnaire (CMDQ) (31). Participants were asked to rate how much ache, pain, or discomfort interfered with work in the last week for 20 body parts. Cronbach’s alphas ranged from .782 to .817 across the two waves.

Stress Self. Participant stress was assessed with a scale of four items, in which respondents answered questions about the extent to which stress affected them at work and home, and healthy management of stress. Cronbach’s alphas ranged from .781 to .792 across the two waves.

Healthy Eating Self. Participant healthy eating habits were assessed with a scale of five items, in which respondents answered questions about making healthy food choices at home and in restaurants. Cronbach’s alphas ranged from .816 to .823 across the two waves.

Physical Activity Habits. Physical activity habits were assessed with an index of four items of the number of days in which respondents performed moderate or heavy exercise for at least 30 minutes, along with a single item assessing the extent to which respondents performed daily exercise for 30 minutes. Cronbach’s alphas ranged from .879 to .888 across the two waves.

Burnout. Burnout was assessed using four items from the Maslach Burnout Inventory (Maslach & Jackson, 1981). Respondents were asked about emotional exhaustion and working too hard. Cronbach’s alphas ranged from .783 to .825 across the two waves.

Alcohol. Alcohol was assessed using seven items from the Health Maintenance Consortium (available at http://hmcrc.srph.tamhsc.edu/Measures/MeasuresMain.html). Cronbach’s alphas ranged from .786 to .810 across the two waves.

Tobacco. Tobacco use was measured by one item, in which respondents answered whether they were ever tobacco users and if so, how frequently and how recently they used tobacco.

Depression. Depression was assessed using five items from the Health Maintenance Consortium (available at http://hmcrc.srph.tamhsc.edu/Measures/MeasuresMain.html). Cronbach’s alphas ranged from .826 to .833 across the two waves.

Table 1 List of Items for each Scales and Index

**Fruits**: single variable, c_ frsrv, is the sum of the 2 calculated fruit daily serving scores.

**Vegetables**: single variable, c_vgsrv, is the sum of the 8 calculated vegetable daily serving scores.

**Fruits/Vegetables**: single variable, c_fvsrv, is the sum of the 10 calculated fruit and vegetable daily serving scores.

**Fat**: *z* scores computed for all 14 items, 11 items reverse coded to reflect higher score = lower fat (3 items not reverse coded are c_sftna, c_sfcrl, and c_sflfc), then 14 items averaged to compute scale.

**Sleep Quality:** *z* scores computed for all 4 items, 2 items reverse coded to reflect higher score = better sleep quality (2 items not reverse coded are slpsat and slp7qly), then 4 items averaged to compute scale.

**Sleep Quantity:** *z* scores computed for all 3 items, items reflect higher score = more sleep, then 3 items averaged to compute scale.

**Fatigue:** *z* scores computed for all 4 items, 3 items reverse coded to reflect higher score = less fatigue (1 item not reverse coded is rsvenrg), then 4 items averaged to compute scale.

**Sf-36: General Health:** all 5 items retain their raw format as all items are in the same metric, 2 items reverse coded to reflect higher score = better general health (3 items not reverse coded are d_sick, d_hlth, and d_worse), then 5 items averaged to compute scale.

**Musculoskeletal Pain (With Feet):** all 20 items retain their raw format as all items are in the same metric, all items reverse coded to reflect higher score = less musculoskeletal pain, then 20 items averaged to compute scale.

**Musculoskeletal Pain (Without Feet):** all 20 items retain their raw format as all items are in the same metric, all items reverse coded to reflect higher score = less musculoskeletal pain, then 18 items averaged to compute scale.

**Stress Self:** all 4 items retain their raw format as all items are in the same metric, 3 items reverse coded to reflect higher score = less stress (item not reverse is imngstr), then 4 items averaged to compute scale.

**Healthy Eating Self:** *z* scores computed for all 5 items, 1 item reverse coded to reflect higher score = healthier eating (4 items not reverse coded are hlthffd, hlthres, me5fv, and melowft), then 5 items averaged to compute scale.

**Physical Activity Self:** *z* scores computed for all 5 items, all items reflect higher score = more physical activity, then 5 items averaged to compute scale.

**Burnout:** all 4 items retain their raw format as all items are in same metric, all items reverse coded to reflect higher score = less burnout, then 4 items averaged to compute scale.

**Alcohol:** *z* scores computed for all 7 items, all items reverse coded to reflect higher score = less alcohol use/issues, then 7 items averaged to compute scale.

**Tobacco**: 1 question, tobac, reverse coded to reflect higher score = absence of/less tobacco use.

**Depression:** all 5 items retain their raw format as all items are in same metric, 3 items reverse coded to reflect higher score = less depression (2 items not reverse coded are depr7dy and deprwk), then 5 items averaged to compute scale.

Table 2 Scale items, names, and response options

| **Question # (for F/V)** | **Variable name** | **Question text** | **Values** | **Construct** |
| --- | --- | --- | --- | --- |
| Q1 | c_juice | Q1-How many times drank juice | 1-Never 2-1 to 3x last month 3-1 to 2x/week 4-3 to 4x/week 5-5 to 6x/week 6-1x/day 7-2x/day 8-3x/day 9-4x/day 10-5 or more x/day | Fruit |
| Q1a | c_juchm | Q1a-How much juice | 1-less than 3/4 c. 2-3/4 to 1 1/4 c. 3-1 1/4 to 2 c. 4-more than 2 c. | Fruit |
| calculated | c_jcfrq | Q1-Times have juice per day |  | Fruit |
| calculated | c_jcamt | Q1-Juice portion size |  | Fruit |
| calculated | c_jcscr | Q1-Juice score (daily avg. servings) |  | Fruit |
| Q2 | c_fruit | Q2-how many times per mo/wk/day did you eat fruit | 1-Never 2-1 to 3x last month 3-1 to 2x/week 4-3 to 4x/week 5-5 to 6x/week 6-1x/day 7-2x/day 8-3x/day 9-4x/day 10-5 or more x/day | Fruit |
| Q2a | c_frthm | Q2a-total amount of fruit per mo/wk/day | 1-less than 1 med fruit or 1/2 c. 2-1 med fruit or 1/2 c. 3-2 med fruits or 1 c. 4-more than 2 med fruits or 1 c. | Fruit |
| calculated | c_ftfrq | Q2-Times have fruit per day |  | Fruit |
| calculated | c_ftamt | Q2-Fruit portion size |  | Fruit |
| calculated | c_ftscr | Q2-Fruit score |  | Fruit |
| Q3 | c_lettc | Q3-how often did you eat lettuce. | 1-Never 2-1 to 3x last month 3-1 to 2x/week 4-3 to 4x/week 5-5 to 6x/week 6-1x/day 7-2x/day 8-3x/day 9-4x/day 10-5 or more x/day | Vegetables |
| Q3a | c_lethm | Q3a-how much lettuce salad. | 1-about 1/2 c. 2-about 1 c. 3-about 2 c. 4-more than 2 c. | Vegetables |
| calculated | c_ltfrq | Q3-Times have lettuce per day |  | Vegetables |
| calculated | c_ltamt | Q3-Lettuce portion size |  | Vegetables |
| calculated | c_ltscr | Q3-Lettuce score (daily avg. servings). |  | Vegetables |
| Q4 | c_frfry | Q4-how often did you eat french fries. | 1-Never 2-1 to 3x last month 3-1 to 2x/week 4-3 to 4x/week 5-5 to 6x/week 6-1x/day 7-2x/day 8-3x/day 9-4x/day 10-5 or more x/day | Vegetables |
| Q4a | c_fryhm | Q4a-how much fries. | 1-smaller order or less 2-medium order 3-large order 4-super size order or more | Vegetables |
| calculated | c_frfrq | Q4-Times have fries per day |  | Vegetables |
| calculated | c_framt | Q4-Fries portion size |  | Vegetables |
| calculated | c_frscr | Q4-French fry score (daily avg. servings). |  | Vegetables |
| Q5 | c_potat | Q5-how often did you eat non fried potatoes. | 1-Never 2-1 to 3x last month 3-1 to 2x/week 4-3 to 4x/week 5-5 to 6x/week 6-1x/day 7-2x/day 8-3x/day 9-4x/day 10-5 or more x/day | Vegetables |
| Q5a | c_pothm | Q5a-how much non fried potatoes. | 1-1 sm or less (1/2 c. or less) 2-1 med (1/2 to 1 c.) 3-1 large (1 to 1 1/2 c.) 4-2 med or more (1 1/2 c. or more) | Vegetables |
| calculated | c_pofrq | Q5-Times have non-fried potatoes per day |  | Vegetables |
| calculated | c_poamt | Q5-Potato portion size |  | Vegetables |
| calculated | c_poscr | Q5-Non-fried potatoes score (daily avg. servings). |  | Vegetables |
| Q6 | c_beans | Q6-how often did you eat cooked dried beans. | 1-Never 2-1 to 3x last month 3-1 to 2x/week 4-3 to 4x/week 5-5 to 6x/week 6-1x/day 7-2x/day 8-3x/day 9-4x/day 10-5 or more x/day | Vegetables |
| Q6a | c_bnshm | Q6a-how much beans. | 1-1/2 c. or less 2-1/2 to 1 c. 3-1 to 1 1/2 c. 4-1 1/2 c. or more | Vegetables |
| calculated | c_bnfrq | Q6-Times have beans per day |  | Vegetables |
| calculated | c_bnamt | Q6-Beans portion size |  | Vegetables |
| calculated | c_bnscr | Q6-Cooked dried beans score (daily avg. servings). |  | Vegetables |
| Q7 | c_oveg | Q7-how often did you eat other vegetables over last month | 1-Never 2-1 to 3x last month 3-1 to 2x/week 4-3 to 4x/week 5-5 to 6x/week 6-1x/day 7-2x/day 8-3x/day 9-4x/day 10-5 or more x/day | Vegetables |
| Q7a | c_veghm | Q7a-total amount vegies each time | 1-1/2 c. or less 2-1/2 to 1 c. 3-1 to 2 c. 4-2 c. or more | Vegetables |
| calculated | c_ovfrq | Q7-times have other veggies per day |  | Vegetables |
| calculated | c_ovamt | Q7-other veggies portion size |  | Vegetables |
| calculated | c_ovscr | Q7-other veggies score |  | Vegetables |
| Q8 | c_tomat | Q18-how often did you eat tomato sauce. | 1-Never 2-1 to 3x last month 3-1 to 2x/week 4-3 to 4x/week 5-5 to 6x/week 6-1x/day 7-2x/day 8-3x/day 9-4x/day 10-5 or more x/day | Vegetables |
| Q8a | c_tomhm | Q8a-total amt.of tomato sauce eaten. | 1-1/4 c. 2-1/2 c. 3-1 c. 4-more than 1 c. | Vegetables |
| calculated | c_tmfrq | Q8-Times have tomato sauce per day |  | Vegetables |
| calculated | c_tmamt | Q8-Tomato sauce portion size |  | Vegetables |
| calculated | c_tmscr | Q8-tomato sauce score (daily avg. servings). |  | Vegetables |
| Q9 | c_vsoup | Q9-how often did you eat vegetable soup. | 1-Never 2-1 to 3x last month 3-1 to 2x/week 4-3 to 4x/week 5-5 to 6x/week 6-1x/day 7-2x/day 8-3x/day 9-4x/day 10-5 or more x/day | Vegetables |
| Q9a | c_vsphm | Q9a-total amt.of vegetable soup eaten. | 1-less than 1 c. 2-1 to 2 c. 3-2 to 3 c. 4-more than 3 c. | Vegetables |
| calculated | c_vsfrq | Q9-Times have vegie soup per day |  | Vegetables |
| calculated | c_vsamt | Q9-Vegie soup portion size |  | Vegetables |
| calculated | c_vsscr | Q9-vegetable soup score (daily avg. servings). |  | Vegetables |
| Q10 | c_vmixt | Q10-how often did you eat mixtures with veggies. | 1-Never 2-1 to 3x last month 3-1 to 2x/week 4-3 to 4x/week 5-5 to 6x/week 6-1x/day 7-2x/day 8-3x/day 9-4x/day 10-5 or more x/day | Vegetables |
| calculated | **c_frsrv** | Avg. servings of fruit per day - sum all fruit scores |  | Fruit |
| calculated | **c_vgsrv** | Avg.servings vegies per day -sum all veg scores |  | Vegetables |
| calculated | **c_fvsrv** | total avg. servings of fruit and veggies per day |  | Fruits/Vegetables |
| 11 | c_sfmay | In last month did you use mayo (not incl fat free) | 1-Never 2-1 to 3x last month 3-1 to 2x/week 4-3 to 4x/week 5-5 to 6x/week 6-1x/day 7-2x/day 8-3x/day 9-4x/day 10-5 or more x/day | Fat |
| 12 | c_sffat | When eating beef, pork, ham, lamb how often eat visible fat | 1-Never 2-Rarely 3-Sometimes 4-Often 5-Almost always | Fat |
| 13 | c_sfskn | When eating poultry how often eat skin (if don't eat poultry choose never) | 1-Never 2-Rarely 3-Sometimes 4-Often 5-Almost always | Fat |
| 14 | c_sfbef | In last month how often did you eat ground beef | 1-Never 2-1 to 3x last month 3-1 to 2x/week 4-3 to 4x/week 5-5 to 6x/week 6-1x/day 7-2x/day 8-3x/day 9-4x/day 10-5 or more x/day | Fat |
| 15 | c_sftna | In last month how often did you eat canned/pkg tuna | 1-Never 2-1 to 3x last month 3-1 to 2x/week 4-3 to 4x/week 5-5 to 6x/week 6-1x/day 7-2x/day 8-3x/day 9-4x/day 10-5 or more x/day | Fat |
| 16 | c_sfcrl | In last month of often eat cold/hot cereal | 1-Never 2-1 to 3x last month 3-1 to 2x/week 4-3 to 4x/week 5-5 to 6x/week 6-1x/day 7-2x/day 8-3x/day 9-4x/day 10-5 or more x/day | Fat |
| 17 | c_sfegg | In last month how often eat eggs (excl egg substitute) | 1-Never 2-1 to 3x last month 3-1 to 2x/week 4-3 to 4x/week 5-5 to 6x/week 6-1x/day 7-2x/day 8-3x/day 9-4x/day 10-5 or more x/day | Fat |
| 18 | c_sfbcn | In last month how often did you eat bacon/sausage | 1-Never 2-1 to 3x last month 3-1 to 2x/week 4-3 to 4x/week 5-5 to 6x/week 6-1x/day 7-2x/day 8-3x/day 9-4x/day 10-5 or more x/day | Fat |
| 19 | c_sfbtr | In last month how often put butter, margarine, sour cream on food | 1-Never 2-1 to 3x last month 3-1 to 2x/week 4-3 to 4x/week 5-5 to 6x/week 6-1x/day 7-2x/day 8-3x/day 9-4x/day 10-5 or more x/day | Fat |
| 20 | c_sfche | In last month how often did you eat cheese | 1-Never 2-1 to 3x last month 3-1 to 2x/week 4-3 to 4x/week 5-5 to 6x/week 6-1x/day 7-2x/day 8-3x/day 9-4x/day 10-5 or more x/day | Fat |
| 21 | c_sfcoo | In last month how often did you eat cookies/cake | 1-Never 2-1 to 3x last month 3-1 to 2x/week 4-3 to 4x/week 5-5 to 6x/week 6-1x/day 7-2x/day 8-3x/day 9-4x/day 10-5 or more x/day | Fat |
| 21a | c_sflfc | When eating cookies/cake, how often low fat | 1-Never 2-Rarely 3-Sometimes 4-Often 5-Almost always | Fat |
| 22 | c_sfice | In last month how often did you eat ice cream or milk shakes | 1-Never 2-1 to 3x last month 3-1 to 2x/week 4-3 to 4x/week 5-5 to 6x/week 6-1x/day 7-2x/day 8-3x/day 9-4x/day 10-5 or more x/day | Fat |
| 23 | c_sfcnd | In lats month how often did you eat candy bars, chocolate candy, toffee | 1-Never 2-1 to 3x last month 3-1 to 2x/week 4-3 to 4x/week 5-5 to 6x/week 6-1x/day 7-2x/day 8-3x/day 9-4x/day 10-5 or more x/day | Fat |
|  | slpsat | In the past 7 days I was satisfied with my sleep | 1-Not at all 2- 3- 4-Somewhat 5- 6- 7-Very much | sleep quality |
|  | slp7qly | In the past week my sleep quality was | 1-Very poor 2- 3- 4-Fair 5- 6- 7-Very good | sleep quality |
|  | slptrbl | In the past 7 days how often have you had trouble sleeping | 0-Never 1-Rarely 2-Sometimes 3-Often 4-Always | sleep quality |
|  | slpqual | During past month how would you rate your sleep quality overall | 0-Very good 1-Fairly good 2-Fairly bad 3-Very bad | sleep quality |
|  | amt | In past month, avg. hours of sleep during each sleep period (taken from vigilance questionnaire) | open-ended | sleep quantity |
|  | slpenuf | In the past 7 days how often have you gotten enough sleep | 0-Never 1-Rarely 2-Sometimes 3-Often 4-Always | sleep quantity |
|  | slpavg | In the past month, how many hours of sleep did you average in a 24 hr period? | 1- Less tha 4 hrs 2- 4-5 hours 3- 6-7 hours 4- 7-8 hours 5- 9 or more hours | sleep quantity |
|  | btwshft | I don't usually get enough time between work shifts to recover my energy fully | 1-Strongly disagree 2-Disagree 3-Slightly disagree 4-No Opinion 5-Slightly agree 6-Agree 7-Strongly agree | fatigue |
|  | exhaust | Physically I feel exhausted today | 1-Strongly disagree 2-Disagree 3-Slightly disagree 4-No Opinion 5-Slightly agree 6-Agree 7-Strongly agree | fatigue |
|  | rsvenrg | I have plenty of reserve energy when I need it | 1-Strongly disagree 2-Disagree 3-Slightly disagree 4-No Opinion 5-Slightly agree 6-Agree 7-Strongly agree | fatigue |
|  | sleepy | In the past 7 days I had a hard time getting things done because I was sleepy | 1-Not at all 2- 3- 4-Somewhat 5- 6- 7-Very much | fatigue |
|  | d_genhl | In general, would you say your health is | 1-Excellent 2-Very good 3-Good 4-Fair 5-Poor | SF-36: general health |
|  | d_sick | how true/false: I seem to get sick easier than other | 1-Definitely true 2-Mostly true 3-Don't know 4-Mostly false 5-Definitely false | SF-36: general health |
|  | d_hlth | how true/false: I am as healthy as anybody | 1-Definitely true 2-Mostly true 3-Don't know 4-Mostly false 5-Definitely false | SF-36: general health |
|  | d_worse | how true/false: I expect my health to get worse | 1-Definitely true 2-Mostly true 3-Don't know 4-Mostly false 5-Definitely false | SF-36: general health |
|  | d_excel | how true/false: my health is excellent | 1-Definitely true 2-Mostly true 3-Don't know 4-Mostly false 5-Definitely false | SF-36: general health |
|  | mdneck | Neck | 1-Not at all 2-Slightly interfered 3-Substantially interfered | Musculoskeletal pain |
|  | mdshldr | Shoulder (Right) | 1-Not at all 2-Slightly interfered 3-Substantially interfered | Musculoskeletal pain |
|  | mdshldl | Shoulder (Left) | 1-Not at all 2-Slightly interfered 3-Substantially interfered | Musculoskeletal pain |
|  | mdupbck | Upper Back | 1-Not at all 2-Slightly interfered 3-Substantially interfered | Musculoskeletal pain |
|  | mduarmr | Upper Arm (Right) | 1-Not at all 2-Slightly interfered 3-Substantially interfered | Musculoskeletal pain |
|  | mduarml | Upper Arm (Left) | 1-Not at all 2-Slightly interfered 3-Substantially interfered | Musculoskeletal pain |
|  | mdlwbck | Lower Back | 1-Not at all 2-Slightly interfered 3-Substantially interfered | Musculoskeletal pain |
|  | mdfarmr | Forearm (Right) | 1-Not at all 2-Slightly interfered 3-Substantially interfered | Musculoskeletal pain |
|  | mdfarml | Forearm (Left) | 1-Not at all 2-Slightly interfered 3-Substantially interfered | Musculoskeletal pain |
|  | mdwrstr | Wrist/Hand (Right) | 1-Not at all 2-Slightly interfered 3-Substantially interfered | Musculoskeletal pain |
|  | mdwrstl | Wrist/Hand (Left) | 1-Not at all 2-Slightly interfered 3-Substantially interfered | Musculoskeletal pain |
|  | mdhip | Hip/Buttocks | 1-Not at all 2-Slightly interfered 3-Substantially interfered | Musculoskeletal pain |
|  | mdthghr | Thigh (Right) | 1-Not at all 2-Slightly interfered 3-Substantially interfered | Musculoskeletal pain |
|  | mdthghl | Thigh (Left) | 1-Not at all 2-Slightly interfered 3-Substantially interfered | Musculoskeletal pain |
|  | mdkneer | Knee (Right) | 1-Not at all 2-Slightly interfered 3-Substantially interfered | Musculoskeletal pain |
|  | mdkneel | Knee (Left) | 1-Not at all 2-Slightly interfered 3-Substantially interfered | Musculoskeletal pain |
|  | mdlwlgr | Lower Leg/Ankle (Right) | 1-Not at all 2-Slightly interfered 3-Substantially interfered | Musculoskeletal pain |
|  | mdlwlgl | Lower Leg/Ankle (Left) | 1-Not at all 2-Slightly interfered 3-Substantially interfered | Musculoskeletal pain |
|  | mdfootr | Foot (Right) | 1-Not at all 2-Slightly interfered 3-Substantially interfered | Musculoskeletal pain |
|  | mdfootl | Foot (Left) | 1-Not at all 2-Slightly interfered 3-Substantially interfered | Musculoskeletal pain |
|  | strwork | I feel significant stress at work | 1-Strongly disagree 2-Disagree 3-Slightly disagree 4-No Opinion 5-Slightly agree 6-Agree 7-Strongly agree | stress self |
|  | strhome | Worrying aobut work issues makes it hard to reax at home | 1-Strongly disagree 2-Disagree 3-Slightly disagree 4-No Opinion 5-Slightly agree 6-Agree 7-Strongly agree | stress self |
|  | strjob | Overall I feel like there is too much stress in my job | 1-Strongly disagree 2-Disagree 3-Slightly disagree 4-No Opinion 5-Slightly agree 6-Agree 7-Strongly agree | stress self |
|  | imngstr | In general I manage stress in a healthy way | 1-Strongly disagree 2-Disagree 3-Slightly disagree 4-No Opinion 5-Slightly agree 6-Agree 7-Strongly agree | stress self |
|  | fastfd | On average how often do you eat fast food | 0-Hardly ever 1-2-3 times a month 2- 2-3 times a week 3-One or more times a day | healthy eating self |
|  | hlthffd | I make healthy choices when ordering fast food | 1-Strongly disagree 2-Disagree 3-Slightly disagree 4-No Opinion 5-Slightly agree 6-Agree 7-Strongly agree | healthy eating self |
|  | hlthres | I select healthy food items whan eating at restaurants | 1-Strongly disagree 2-Disagree 3-Slightly disagree 4-No Opinion 5-Slightly agree 6-Agree 7-Strongly agree | healthy eating self |
|  | me5fv | In general I eat 5+ servings of f/v per day | 1-Strongly disagree 2-Disagree 3-Slightly disagree 4-No Opinion 5-Slightly agree 6-Agree 7-Strongly agree | healthy eating self |
|  | melowft | In general I eat a low-fat diet | 1-Strongly disagree 2-Disagree 3-Slightly disagree 4-No Opinion 5-Slightly agree 6-Agree 7-Strongly agree | healthy eating self |
|  | exhrd | How many days per wk did you exercise or take part in hard physical activites | 0-7 days per wk | physical activity self |
|  | exmodr | How many days per wk did you exercise or take part in moderate physical activites | 0-7 days per wk | physical activity self |
|  | exstrng | How many days per week did you execise to strengthen or tone muscles | 0-7 days per wk | physical activity self |
|  | exsweat | In a typical wk how many days do you take part in any phys activity long enough to work up a sweat | 0-7 days per wk | physical activity self |
|  | ex30m | I exercise for 30 minutes almost every day | 1-Strongly disagree 2-Disagree 3-Slightly disagree 4-No Opinion 5-Slightly agree 6-Agree 7-Strongly agree | physical activity self |
|  | drained | I feel emotionally drained from my work | 1-Strongly disagree 2-Disagree 3-Slightly disagree 4-No Opinion 5-Slightly agree 6-Agree 7-Strongly agree | burn out |
|  | toohard | I feel I'm working too hard on my job | 1-Strongly disagree 2-Disagree 3-Slightly disagree 4-No Opinion 5-Slightly agree 6-Agree 7-Strongly agree | burn out |
|  | burnout | I feel burned out from my work | 1-Strongly disagree 2-Disagree 3-Slightly disagree 4-No Opinion 5-Slightly agree 6-Agree 7-Strongly agree | burn out |
|  | usedup | I feel used up at the end of the workday | 1-Strongly disagree 2-Disagree 3-Slightly disagree 4-No Opinion 5-Slightly agree 6-Agree 7-Strongly agree | burn out |
|  | alcone | In past 30 days how frequently do you have at least one alcoholic drink | 0-Never 1-About once a month 2-Several times per month 3-About once a week 4-Several times per week 5-Almost every day | alcohol |
|  | alcsevl | In past 30 days how frequently do you have several drinks in one sitting | 0-Never 1-About once a month 2-Several times per month 3-About once a week 4-Several times per week 5-Almost every day | alcohol |
|  | alcbuzz | In past 30 days how frequently do you drink to the point of getting buzzed | 0-Never 1-About once a month 2-Several times per month 3-About once a week 4-Several times per week 5-Almost every day | alcohol |
|  | alcdrnk | in past 30 days how frequently do you drink to the point of getting drunk | 0-Never 1-About once a month 2-Several times per month 3-About once a week 4-Several times per week 5-Almost every day | alcohol |
|  | alcglt | How often during last 30 days have you had feelings of guilt or remorse after drinking | 0-Never 1-Once 2-Two or three times 3-Weekly 4-More than once a week | alcohol |
|  | alfam | In the last 6 mo, my family or friends have been concerned with my drinking | 1-Strongly disagree 2-Disagree 3-Slightly disagree 4-No Opinion 5-Slightly agree 6-Agree 7-Strongly agree | alcohol |
|  | alcquit | In the last 6 mo, I have tried to quit drinking | 1-Strongly disagree 2-Disagree 3-Slightly disagree 4-No Opinion 5-Slightly agree 6-Agree 7-Strongly agree | alcohol |
|  | tobac | Do you currently use tobacco (cigarettes, cigars, pipe or chewing tobacco) Mark one. | 0-No and never was regular user 1-No and quit more than 6 mo ago 2-No and quit less than 6 mo ago 3-Yes current user (1 pack/day or less) 4-Yes, current user (>1 pack/day) | tobacco |
|  | happymo | Overall in the last month I have been happy | 1-Strongly disagree 2-Disagree 3-Slightly disagree 4-No Opinion 5-Slightly agree 6-Agree 7-Strongly agree | depression |
|  | happywk | I feel happy to go to work each day | 1-Strongly disagree 2-Disagree 3-Slightly disagree 4-No Opinion 5-Slightly agree 6-Agree 7-Strongly agree | depression |
|  | depr7dy | In the past 7 days, I felt depressed | 1-Strongly disagree 2-Disagree 3-Slightly disagree 4-No Opinion 5-Slightly agree 6-Agree 7-Strongly agree | depression |
|  | deprwk | Feelings of depression interfere with my work | 1-Strongly disagree 2-Disagree 3-Slightly disagree 4-No Opinion 5-Slightly agree 6-Agree 7-Strongly agree | depression |
|  | jobdepr | Overall my job makes me feel depressed | 1-Strongly disagree 2-Disagree 3-Slightly disagree 4-No Opinion 5-Slightly agree 6-Agree 7-Strongly agree | depression |
|  |  |  |  |  |
|  |  |  |  |  |
|  |  |  |  |  |
|  |  |  |  |  |
|  |  |  |  |  |
|  |  |  |  |  |
|  |  |  |  |  |
|  |  |  |  |  |
|  |  |  |  |  |
|  |  |  |  |  |
|  |  |  |  |  |

Table 3. Baseline and 6-month follow-up Reliability of Constructs Measured in Questionnaire

| **Cronbach α** | | | | | |
| --- | --- | --- | --- | --- | --- |
| **Construct** | **Baseline** | | **6 Month Follow-up** | | **Test-Retest** |
|  | **Raw** | **Standardized** | **Raw** | **Standardized** | **Correlation** |
| Fruit Consumption* | 0.048 | 0.057 | 0.381 | 0.431 | 0.485 |
| Vegetable Consumption* | 0.428 | 0.526 | 0.390 | 0.494 | 0.480 |
| Total Fruit and Vegetable consumption* | 0.499 | 0.533 | 0.465 | 0.521 | 0.554 |
| Fat Consumption | 0.585 | 0.585 | 0.634 | 0.638 | 0.696 |
| Sleep Quality | 0.914 | 0.914 | 0.908 | 0.907 | 0.652 |
| Sleep Quantity | 0.820 | 0.817 | 0.811 | 0.811 | 0.652 |
| Fatigue | 0.687 | 0.686 | 0.711 | 0.711 | 0.645 |
| Karolinska Sleepiness Scale | N/A | N/A | N/A | N/A | 0.292 |
| SF36: General Health | 0.770 | 0.767 | 0.764 | 0.761 | 0.743 |
| Musculoskeletal Pain (20 items) | 0.782 | 0.789 | 0.817 | 0.817 | 0.635 |
| Musculoskeletal Pain (18 items) | 0.774 | 0.783 | 0.802 | 0.801 | 0.630 |
| Stress Self | 0.781 | 0.781 | 0.792 | 0.788 | 0.732 |
| Healthy Eating Self | 0.823 | 0.823 | 0.816 | 0.817 | 0.733 |
| Physical Activity | 0.888 | 0.887 | 0.879 | 0.882 | 0.632 |
| Burnout | 0.783 | 0.783 | 0.825 | 0.825 | 0.654 |
| Alcohol | 0.810 | 0.809 | 0.786 | 0.790 | 0.835 |
| Tobacco | N/A | N/A | N/A | N/A | 0.909 |
| Depression | 0.833 | 0.834 | 0.826 | 0.829 | 0.701 |

* The construct is an index and Cronbach's α is not interpreted as a measure of internal consistency because individual items are summed to form the index.
